# Identification of a contemporary human parechovirus type 1 by VIDISCA and characterisation of its full genome

**DOI:** 10.1186/1743-422X-5-26

**Published:** 2008-02-12

**Authors:** Luciano Kleber de Souza Luna, Sigrid Baumgarte, Klaus Grywna, Marcus Panning, Jan Felix Drexler, Christian Drosten

**Affiliations:** 1 Clinical Virology Group, Bernhard Nocht Institute for Tropical Medicine, Hamburg, Germany; 2 Laboratory of Virology, Department of Microbiological Consumer Protection, Institute of Hygiene and the Environment, Hamburg, Germany; 3 Institute of Virology, University of Bonn Medical Centre, Sigmund Freud-Str. 25, 53127 Bonn, Germany

## Abstract

**Background:**

Enteritis is caused by a spectrum of viruses that is most likely not fully characterised. When testing stool samples by cell culture, virus isolates are sometimes obtained which cannot be typed by current methods. In this study we used VIDISCA, a virus identification method which has not yet been widely applied, on such an untyped virus isolate.

**Results:**

We found a human parechovirus (HPeV) type 1 (strain designation: BNI-788st). Because genomes of contemporary HPeV1 were not available, we determined its complete genome sequence. We found that the novel strain was likely the result of recombination between structural protein genes of an ancestor of contemporary HPeV1 strains and nonstructural protein genes from an unknown ancestor, most closely related to HPeV3. In contrast to the non-structural protein genes of other HPeV prototype strains, the non-structural protein genes of BNI-788st and HPeV3 prototype strains did not co-segregate in bootscan analysis with that of other prototype strains.

**Conclusion:**

HPeV3 nonstructural protein genes may form a distinct element in a pool of circulating HPeV non-structural protein genes. More research into the complex HPeV evolution is required to connect virus ecology with disease patterns in humans.

## Background

The *Picornaviridae *are a highly diversified family of non-envevloped plus-strand RNA viruses, many of which are pathogenic for humans. Their full genetic and phenotypic spectrum is unknown and novel picornavirus strains keep being discovered [1,2]. Large work has been invested in recent years in the development of methods for discovering new and unknown viruses. Sophisticated approaches, such as highly redundant cDNA arrays, high-throughput cDNA library analysis, and ultradeep sequencing have been successfully used [3-7]. These methods are expensive and require expert knowledge, prohibiting their use in general diagnostic laboratories.

A simpler method, termed Virus Discovery cDNA AFLP (VIDISCA), uses cell culture supernatants treated by DNase digestion in a modified cDNA Amplified Fragment Length Polymorphism (AFLP) analysis. AFLP employs restriction enzyme digestion sites in an unknown DNA sequence to ligate oligonucleotide adaptors, which are then used as primer binding sites for PCR amplification. This method has been described originally in the context of the discovery of a novel human Coronavirus in 2004 [8]. In that study, it was used to amplify an untypable virus from the supernatant of a cell culture showing a cytopathic effect (CPE).

As CPE-positive but serologically untypable cell cultures occur regularly during routine diagnostics, it would be desirable to have a simple and inexpensive method for the characterisation of viruses from supernatants. VIDISCA seems to be an interesting option, even though the procedure has not been employed by other groups after its original description [8]. It is unclear whether it can be adapted for routine use from the literature and whether it is practically useful.

In this study, we adapted VIDISCA with slight modifications and applied it on a cytopathic cell-culture obtained during routine surveillance of human enteritis. From the culture we amplified fragments of what turned out to be a human parechovirus type 1. Parechoviruses form a separate genus within the family *Picornaviridae*. Members of the species Human Parechovirus (HPeV) cause symptoms of common cold and enteritis, but also encephalitis, myocarditis, and other conditions [9]. Until their reclassification HPeV types 1 and 2 have been known as Echovirus types 22 and 23, within the Enterovirus genus. Very recently, four novel HPeV types have been described, fully sequenced, and intensively studied [9-16]. Genome data on HPeV type 1, however, have not been updated after the genome of the prototype strain was characterised [17]. This strain was isolated in the 1960s. For more recent strains, only limited sequencing of a small part of the structural protein gene P1 has been done. Because recent studies suggested that HPeV 1 may have undergone significant evolution including recombination with other strains [14,16], the full genome sequence of the type 1 HPeV identified in this study was determined and analysed for recombination. We found evidence of the novel strain resulting from non-recent recombination between HPeV1 structural protein genes and non-structural protein genes of another type, potentially type 3. This was probably followed by another recombination within the structural protein genes of contemporary type 1 viruses.

## Results

**Figure 1:**
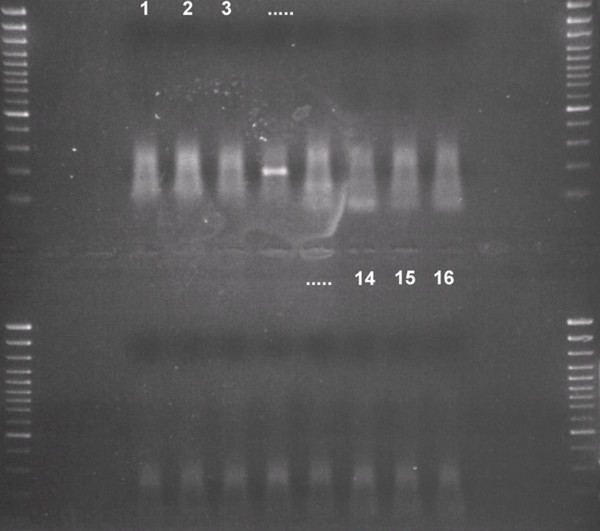
Original agarose gel photograph obtained from second amplification step of VIDISCA. Numbering of the 16 second-stage PCR products is shown above the first and last three lanes. The product in lane 4 was sequenced and showed a parechovirus as described in the text. A 100 bp marker is used on the gel (500 bp band enhanced, 400, 300, 200, 100 bp).

During routine diagnostic work on patients with acute enteritis in a municipal health service, a stool sample from a 30 year-old female kitchen worker with acute enteritis displayed a cytopathic effect (CPE) on cultured African Green Monkey Kidney (GMK) cells. The CPE resembled that of enteroviruses, including rounding and blebbing, shrinking, and detachment of cells from the monolayer. The virus isolate could be passaged to uninfected cells but showed no detectable neutralisation if subcultured with several different pools of polyclonal anti-enterovirus sera. RT-PCR for Norovirus and Enterovirus, PCR for Adenovirus, and antigen-EIA for Astro- and Rotavirus were negative on the supernatant and on the original patient material. The unknown isolate was termed BNI-788st. In order to type it, supernatant was subjected to VIDISCA, with an additional ultracentrifugation step as opposed to the original protocol [8]. In the second amplification stage, one of 16 PCR reactions yielded a distinct amplification product (Figure 1). Sequencing showed a 188 nucleotide DNA fragment that was homologous in a nucleotide BLAST search with the capsid (P1) protein region of HPeV strains. It should be noted here that no specific Parechovirus diagnostics (serotyping of cell culture, RT-PCR) had been done because these viruses are known to occur almost exclusively in children, and this patient was an adult.

**Figure 2:**
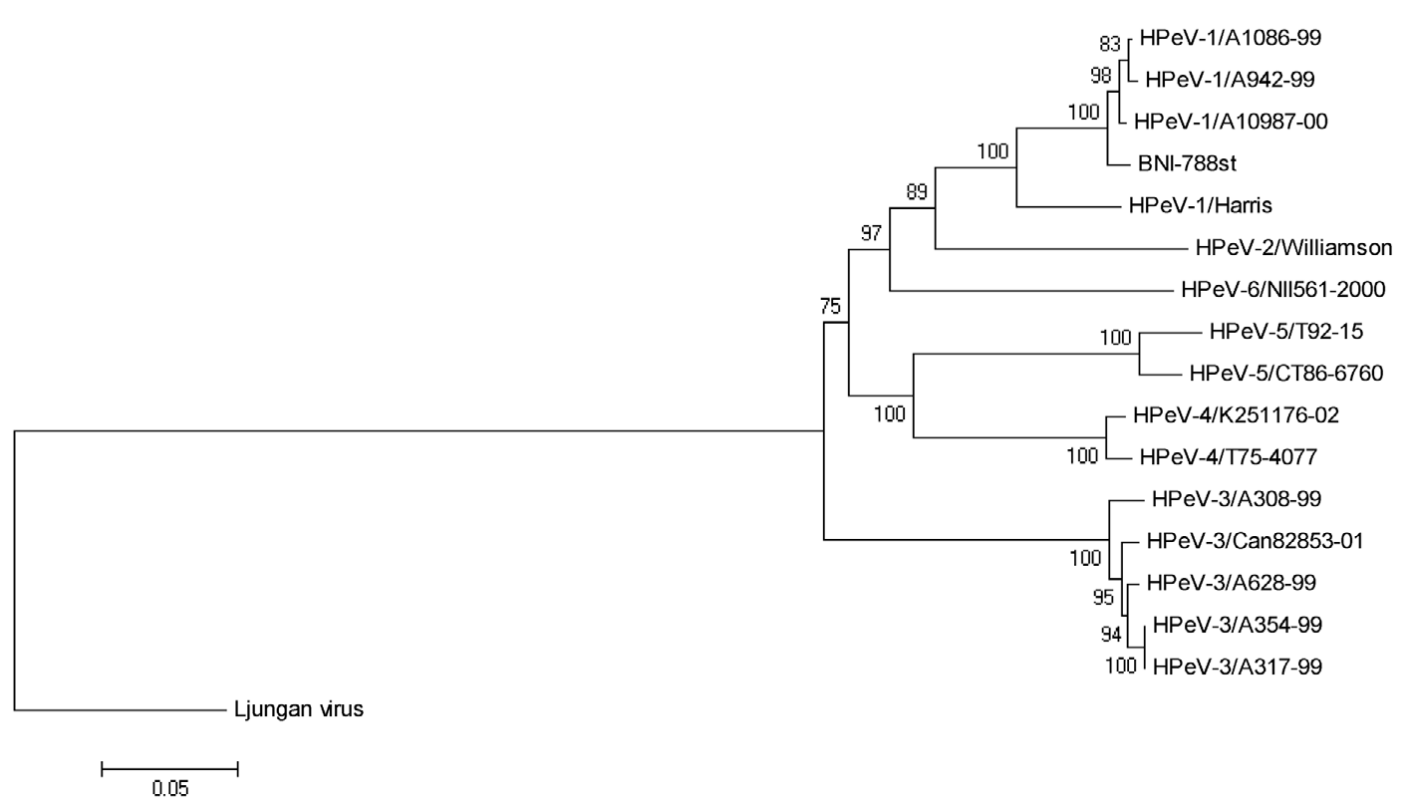
Phylogenetic analysis of P1 protein regions (amino acids 76–773, isolate Harris), analysed using the p-distance substitution model. VP1-based HPeV-types are shown next to clades on the right margin. Analysis was conducted in MEGA4 [42]. The evolutionary histories were inferred using the Neighbor-Joining method [44]. Relevant boostrap values from 500 replicate trees are shown next to the branches [45]. The scale shows evolutionary distance from each root. GenBank accession number of strain BNI-788st is EF051629. The tree was rooted against Ljungan virus, a murine parechovirus.

The VP1 protein gene of BNI-788st was determined as described in [16]. Phylogenetic analysis showed that it clustered with that of a group of contemporary HPeV1 strains (Figure 2). As observed earlier [14], the prototype Echovirus 22 strain Harris had only basal relationship with these strains. Amino acid identity with prototype strain Harris was around 92%. Because no full sequence of any contemporary HPeV1 was available in GenBank, the complete genome sequence of BNI-788st was analysed. Genome length was 7337 nucleotides excluding the poly(A) tract. Genome organization matched that of other parechoviruses, including a 5' untranslated region (UTR) of 709 nucleotides, followed by a large open reading frame of 6537 nucleotides that encoded the putative polyprotein precursor of 2179 amino acids; and a 3' UTR of 91 nucleotides followed by a poly(A) tail.

**Figure 3:**
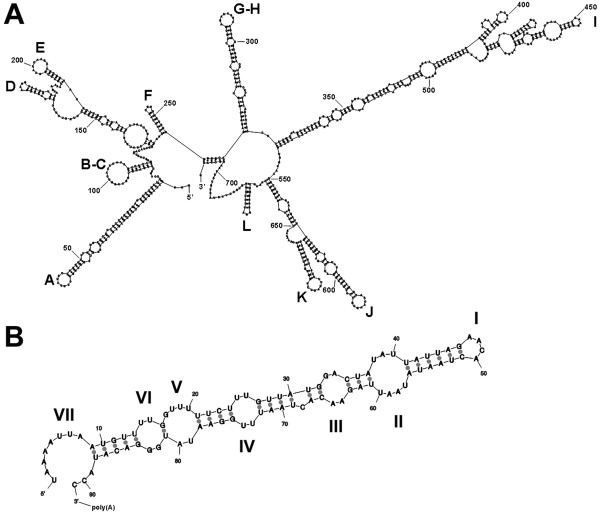
Predicted secondary structure elements of the 5'-noncoding region (A) and 3'-noncoding region (B). Capital letters as used in [19] denominate structural elements of the 5'-noncoding region. Latin numbers for the 3'-nonconding region are used in analogy with [11].

Interestingly, the 5' UTR was most similar to that of HPeV4 viruses, showing 88.9% and 90.8% nucleotide identity with type 4 prototype strains T75-4077 and K251176-02, respectively. The predicted RNA secondary structure elements of the 5' UTR of BNI-788St are depicted in Figure 3. The shown structures were selected from alternative energetically possible structures to resemble most closely the structures of HPeV1 Harris and HPeV2 Williamson [18,19]. Differences were observed in stem-loop elements B, C, G and H. In BNI-788st, corresponding sequences formed only 2 stem-loops, designated B-C and G-H. The other stem-loop elements were well conserved, including I to L which form a type II internal ribosome entry site (IRES) as described for cardioviruses and aphthoviruses [20-22]. A polypyrimidine-rich tract typical of picornaviruses was present 17 nucleotides upstream of the initiation codon, including a Kozak sequence (ANNAUGG).

The predicted secondary structure of the 3' UTR of BNI-788st is also shown in Figure 3. The conformation in terms of relative sizes of loop structures was more similar to HPeV 3 protype strains than to the HPeV 1 prototype strain ([11]). The region was organised in one continuous stem-loop element as recently described for HPeV 1–3 [11]. This was in contrast to other enteroviruses whose 3'-noncoding regions form 2 to 3 such stem loops [23]. A conserved repeat structure as recently described for prototype HPeV [14] was also present.

The genes coding for structural proteins VP0, VP3 and VP1 were most similar to HPeV1, as listed in Table 1. An RGD motif as present in all HPeV except HPeV 3 was present [10,12]. This element is critical in attachment and entry into host cells in other picornaviruses [24] and has been shown to be essential for infectivity in HPeV [25,26]. All of the genes coding for the non-structural proteins were more similar to HPeV3 than to HPeV 1, 2, 4, 5, or 6. Conserved elements such as the 2C helicase motifs GXXGXGK(S/T) and DDLXQ, the 3C protease active-site motif GXCG, and the 3D RNA-dependent RNA polymerase motifs YGDD, KDELR, PSG, and FLKR were all confirmed.

**Table 1:** Nucleotide and amino acid identity with reference strains, by virus protein

**Nucleotide Identity (amino acid identity)**										
Region	HPeV1^a^	HPeV2^b^	HPeV3^c^	HPeV3^d^	HPeV4^e^	HPeV4^f^	HPeV5^g^	HPeV5^h^	HPeV6^i^	HPeV6^j^

5'UTR	86.6	84.9	84.9	85.2	88.9	90.8	ND*	ND	88.3	ND
VP0	82.6 (94.1)	75.7 (84.1)	70.6 (73.4)	71.0 (73.4)	72.9 (78.9)	72.9 (79.2)	72.3 (79.2)	72.3 (79.2)	72.7 (78.9)	72.3 (79.2)
VP3	76.9 (89.3)	72.8 (82.2)	68.1 (76.3)	67.9 (76.7)	71.7 (79.8)	70.2 (80.2)	68.9 (76.7)	68.5 (77.1)	73.1 (83.4)	72.1 (83.8)
VP1	76.6 (89.6)	71.1 (79.2)	64.2 (70.6)	64.6 (71.0)	70.7 (74.0)	71.7 (77.1)	69.6 (74.0)	65.7 (71.4)	69.7 (74.9)	69.3 (74.9)
2A	76.7 (89.3)	77.1 (87.3)	87.1 (88.0)	88.7 (87.3)	76.2 (89.3)	88.2 (94.7)	79.1 (88.7)	76.2 (88.7)	79.8 (88.7)	78.2 (88.7)
2B	82.2 (96.7)	78.4 (95.9)	91.5 (100.0)	91.0 (100.0)	83.1 (100.0)	85.2 (100.0)	83.9 (98.4)	83.3 (98.4)	82.0 (97.5)	82.8 (98.4)
2C	79.4 (92.7)	77.4 (86.6)	92.8 (99.1)	92.0 (98.8)	82.1 (97.3)	88.1 (98.2)	81.8 (95.1)	79.3 (93.9)	78.6 (91.8)	80.1 (96.4)
3A	75.8 (87.2)	76.9 (83.8)	91.2 (91.5)	92.6 (94.0)	81.8 (93.2)	83.2 (92.3)	80.1 (81.2)	77.5 (88.9)	78.6 (88.0)	80.3 (91.5)
3B	71.7 (90.0)	70.0 (90.0)	86.7 (85.0)	86.7 (90.0)	78.3 (95.0)	73.3 (85.0)	80.0 (90.0)	66.7 (90.0)	78.3 (90.0)	76.7 (90.0)
3C	81.0 (97.0)	81.7 (97.0)	91.0 (98.0)	91.3 (98.5)	86.8 (98.0)	87.0 (97.5)	79.5 (99.0)	80.5 (96.0)	84.2 (98.0)	81.3 (97.0)
3D	82.8 (95.9)	82.9 (94.2)	91.0 (97.0)	90.8 (97.0)	88.7 (97.0)	87.9 (97.7)	82.3 (95.1)	82.4 (93.8)	83.9 (95.7)	82.3 (95.3)
3'UTR	83.3	87.9	94.5	92.3	89.0	94.5	84.6	89.0	83.5	84.6

Strain designations: ^a^Harris,^b^Williamson,^c^A308/99,^d^Can82853-01,^e^T75-4077,^f^K251176-02,^g^CT86-6760, ^h^T92-15, ^i^NII561-2000, ^j^BNI-67, *ND = not determined.

**Figure 4:**
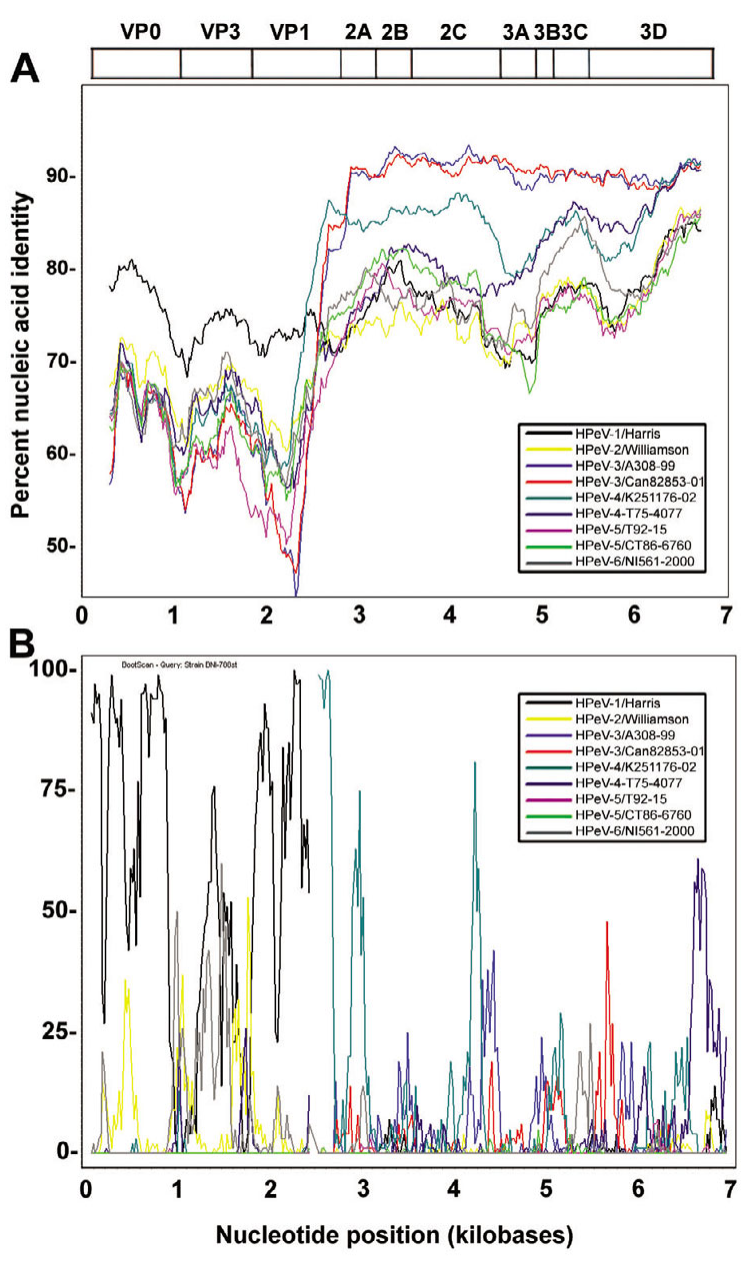
Similarity plot analysis. Analysis was carried out with SimPlot software [43], using a 200 bp sliding window and 50 bp step size. Prototype strains used for comparison are shown in the insert window. A, nucleic acid identity, per analysis window, for strain BNI-788st with prototype strains. Nucleotide positions on x-axis show the centre of the window. B, Bootsan analysis using the same window settings. A bootstrapped phylogenetic analysis is conducted per window over the alignment. Graphs represent the percentage (bootstrap values) at which each strain co-segregates phylogenetically in the analysis window with strain BNI-788st.

As suggested from above results, as well as from similarity values listed in Table 1, the protein-coding genes of BNI-788st might result from recombination between HPeV1 and another HPeV type, potentially type 3. To investigate this further, similarity plot analysis on the full polyprotein open reading frame was conducted as shown in Figure 4. Structural proteins had closest identity with HPeV1 (see below). The non-structural protein portion was 87–92.8% identical to HPeV3 (Table 1), which is less than the degree of identity between HPeV3 prototype strains (96–99%) but more than between prototype strains of non-homologous types (always below 87%, Table 1 and data not shown).

Bootscan analysis was done next. Within the structural gene portion, analysis yielded co-segregation values between BNI-788st and the prototype HPeV1 strain Harris in the order of 95% in VP0 and 90% in VP1. For VP3, a maximum co-segregation value of 70% could be identified only in a very small part of the protein. An abrupt halt of co-segregation with the HPeV1 prototype was observed beyond the VP1 protein portion by bootscan analysis. At the VP1/2A border a crossing point with HPeV4 was identified by the software, but the region in which co-segregation occurred was rather short (Figure 4). Over the rest of the non-structural protein gene, no relevant evidence of recombination with any other HPeV type was observed. Thus, only the degree of nucleotide identity (see above) suggests that the closest relative in the non-structural protein gene portion may be an HPeV3 strain.

**Figure 5:**
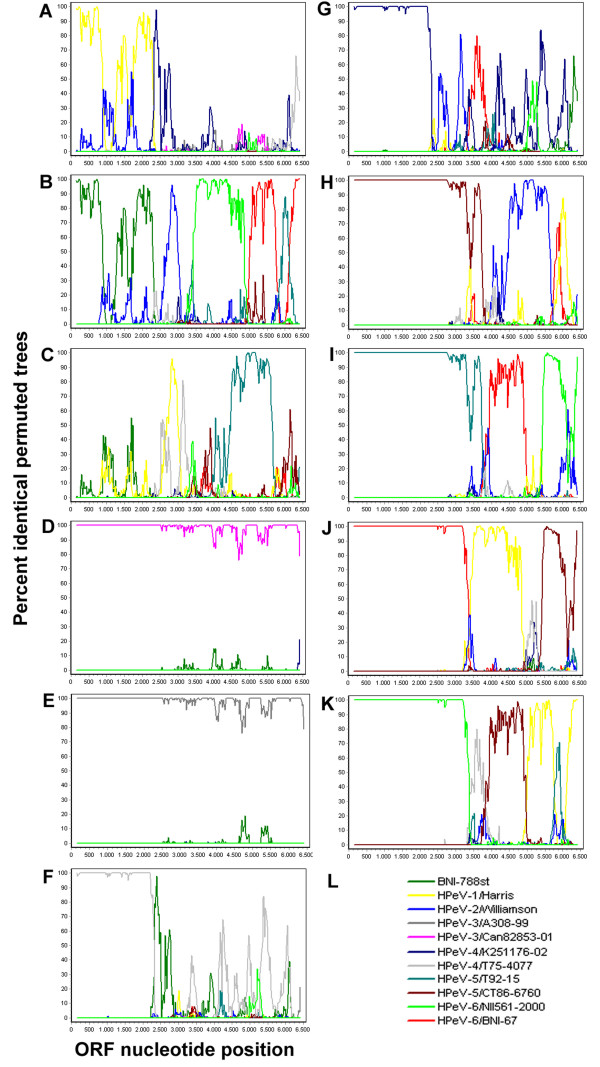
Bootscan analysis as in Figure 4, employing prototype HPeV strains as query sequences. A, BNI-788st (note a slight difference in co-segregation graph as opposed to Figure 4, due to a larger sliding window size of 600 bp in this analysis); B, HPeV-1 prototype strain Harris; C, HPeV-2 prototype strain Williams; D, HPeV-3 prototype strain A308-99; E, HPeV-3 prototype strain Can82853-01; F, HPeV-4 prototype strain K251176-02; G, HPeV-4 prototype strain T75-4077; H, HPeV-5 prototype strain T92-15; I, HPeV-5 prototype strain CT86-6760; J, HPeV-6 prototype strain NII-561-2000; K, HPeV-6 prototype strain BNI-67; L, color legend. In each panel, each coloured graph represents the degree of phylogenetic co-segregation with the query type in a 600 bp sliding analysis window, centered around the nucleotide position given on the x-axis. A bootstrapped phylogenetic analysis was conducted every 50 bp.

For comparison, bootscan analysis was repeated using each of the reference strains for HPeV types 1–6 as the comparison sequence (Figure 5). Most of them showed significant co-segregation with other reference strains alternating over parts of their non-structural genes. Such an observation would be compatible with mosaic recombination in the non-structural genes. No reference strain showed recombination with BNI-788st. Similarly, no indication of past recombination with relatives of other prototypes was seen in the HPeV3 reference strains (see Figure 5, panels D and E).

**Figure 6:**
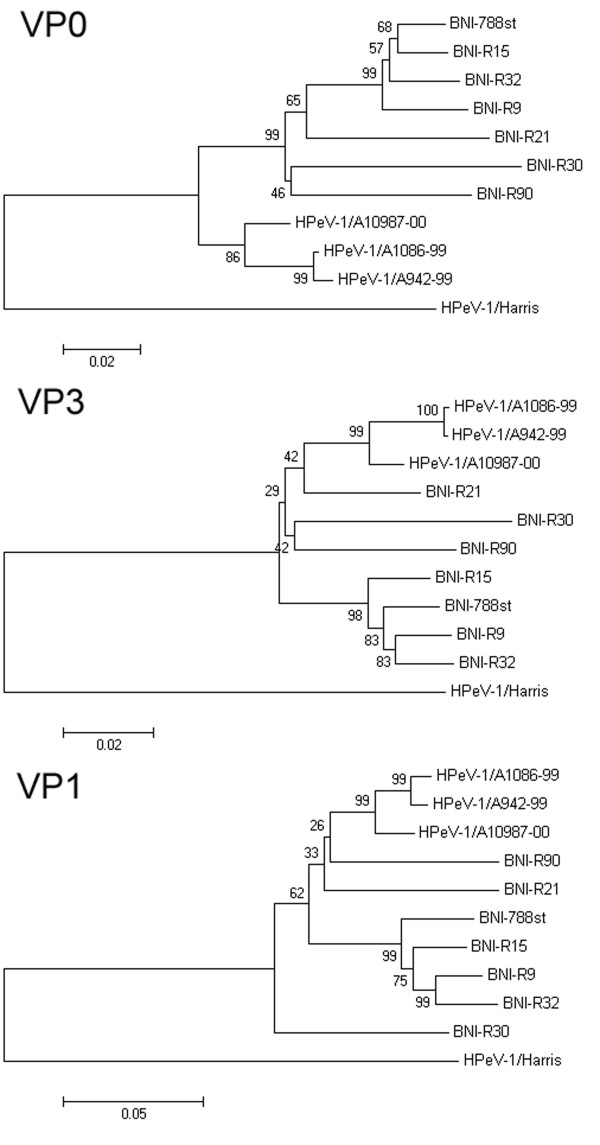
Phylogenetic analysis of the three functional elements of the structural proteins, analysed from amino acid sequences using the p-distance substitution model. Analysis was conducted in MEGA4 [42]. The evolutionary histories were inferred using the Neighbor-Joining method [44]. Boostrap values from 500 replicate trees are shown next to the branches [45]. The scale shows evolutionary distance from each root. GenBank accession numbers: [BNI-67, GenBank: EU022171; BNI-788st, GenBank: EF051629; BNI-R90, GenBank: EU024630; BNIR4, GenBank: EU024631; BNI-R9, GenBank: EU024632; BNI-R15, GenBank: EU024633; BNI-R21, GenBank: EU024634; BNI-R30, GenBank: EU024635; BNI-R32, GenBank: EU024636].

To appreciate in more detail the composition of structural protein genes of BNI-788st, these were compared phylogenetically with that of other contemporary viruses co-circulating in Germany as identified in a recent study [16]. As shown in Figure 6, there was a group of related viruses (BNI-788st, R9, R15, R32) whose VP3 portions were directly originating from the root point of contemporary type 1 viruses, suggesting that they stemmed directly from a common ancestor of all contemporary type 1 viruses. Other circulating strains from Germany (BNI- R21, R30, R90) and Japan [10] formed separate evolutionary lineages. For VP1 the same group of viruses related to BNI-788st existed, but one strain (BNI-R30) was placed between this group and the common ancestor of contemporary strains. BNI-R30 may thus have obtained its VP1 protein earlier than the 788st-related viruses from a common source. Nevertheless, for the 788st-related group (BNI-788st, R9, R15, R32) the length of the internal branch leading to its basal node suggests that their VP1 has been obtained from a non-recent ancestor common to these and most other contemporary HPeV1. In VP0 the BNI-788st-related group was not so close to the root of contemporary strains, suggesting that VP0 may have been acquired by a more recent ancestor of the group by recombination.

## Discussion

Enteritis is caused by a spectrum of viruses that is most likely not fully characterised. When testing stool samples by cell culture, virus isolates are sometimes obtained which cannot be typed by current methods. In this study we confirmed that VIDISCA [8], a virus identification method which has not yet been widely applied, is capable of identifying novel viruses grown in cell culture. We found a contemporary HPeV type 1 strain and analysed its full genome.

The targeted technical search for novel viral agents has become a focus in virology, triggered by the identification of important new agents such as human herpesvirus type 8 [27], human metapneumovirus [28], and SARS-Coronavirus [29]. More recently identified agents include the human coronaviruses NL63 [8] and HKU1 [30], human bocavirus [6], as well as polyomaviruses WU [31] and KI [4]. Different technical approaches have been followed to find novel viruses, including whole virus genome microarrays [3], cDNA-libraries [5], as well as ultra deep sequencing approaches [7]. All of these methods are too sophisticated and costly for routine application.

Using a combination of protected nuclease digestion and AFLP-PCR, van der Hoek et al. have developed VIDISCA as an alternative approach to identifying unknown viruses, at least if they are growing in cell culture [8]. By applying VIDISCA independently, this study proves that the assay is applicable and can be reproduced easily from the literature. The procedure employed a combination of ready-to-use molecular biology reagents that could be used without technical difficulties. The entire procedure including virus particle enrichment, nuclease digestion, nucleic acids preparation, double stranded cDNA synthesis, restriction digestion, adapter ligation and two stages of PCR amplification took two full working days to be completed. Hands-on time for one full staff member was about one working day.

The finding of evidence for a potentially recombinant ancestry of our contemporary HPeV1 strain is rather interesting. HPeV1 and 2, formerly classified as echovirus types 22 and 23 [32], were described in the 1960s. Recent intensified molecular surveillance has revealed HPeV3 [9-12], HPeV4 [13], HPeV5 [14], and HPeV6 [15,16] in very short sequence. However, no further studies of full genomes of currently circulating isolates of HPeV1 have been conducted. A finding of recombination in principle is not surprising given the propensity of picornaviruses [33-37] including parechoviruses [14], to recombine. Following an accepted notion, genomes of the related enteroviruses form a pool of circulating non-structural gene elements that maintain themselves in the host population by recombination with structural protein genes from other members of their genus [35,36]. Our contemporary strain was probably derived from a recombinant ancestor, with a breakpoint at the border between structural and non-structural genes. Most parts of the structural genes were similar to HPeV1, while the non-structural genes were more similar to that of HPeV3. The 5'-noncoding elements were probably contributed by HPeV4.

The non-structural protein genes of BNI-788st were most similar to those of HPeV3, and it is interesting that only BNI-788st and both HPeV3 prototype strains did not show co-segregation of their non-structural genes with that of other prototype strains in bootscan analysis. Within the above-mentioned hypothesis, it would be conceivable that HPeV3 non-structural protein genes could form more inert elements within the pool of HPeVs that may not easily recombine with non-structural genes of other HPeV. Together with our analysis of phylogeny and recombination patterns, this special feature makes it possible to reconstruct likely events in the formation of BNI-788st.

Phylogenetic analysis of the whole non-structural gene portion placed BNI-788st and both HPeV3 strains behind a common ancestor with 88% bootstrap support. This common ancestor would have accepted a complete set of structural protein genes by recombination in the 5'-proximal part of the non-structural protein genes, close to the VP1/2A border. Because the VP3 portion of BNI-788st and its group of relatives is directly derived from the common ancestor of VP3 proteins of all contemporary strains, this recombination would have been a basal, non-recent event. The same can be confirmed in the VP1 portion, where the 788st group is in basal position related to the other contemporary type 1 viruses. It should nevertheless be mentioned that BNI-R30 seems to have taken its VP1 protein from an even older ancestor that is not preserved in other contemporary type 1 strains and has also been lost in BNI-R30 in the other structural protein portions. As a more recent event in the formation of BNI-788st, the common ancestor of the BNI-788st-related group would have acquired its VP0 region from a contemporary type 1 strain. Such intra-capsid recombination in picornaviruses is an uncommon event [34], but yet it has been described for several picornaviruses including Foot-and-Mouth Disease Virus [38], poliomyelitis virus type 1 [39], human enterovirus species B [40], and hepatitis A virus [41].

As a final step, the 5'-noncoding region of BNI-788st could have been acquired from an HPeV4, as suggested from the analysis of its predicted structural properties. Such recombination is frequently observed in other picornaviruses [34]. However, it cannot be analysed from available data whether this has occurred before or along with acquisition of VP0. The secondary structure prediction of the 5'-noncoding region will help following this up, as soon as more 5'-noncoding region sequences of HPeV 1 and HPeV4 will have been characterised.

## Conclusion

In conclusion, this contemporary HPeV1 strain provides limited but plausible evidence of recombination between type 3 nonstructural protein genes and type 1 structural protein genes, which has not been observed before. The surprising absence of mosaic recombination in the non-structural protein genes of BNI-788st and of HPeV3 prototype strains underlines the lack of knowledge on the genesis and ecology of human parechoviruses. More research into parechovirus genome diversity is necessary in order to connect virus ecology with disease patterns in humans.

## Materials and Methods

### Patients and samples

A cell culture supernatant from GMK-AH1 cells (African green monkey kidney cells) which showed a cytopathogenic effect (CPE) was obtained during routine testing for agents of viral enteritis.

### VIDISCA

Virus discovery cDNA-Amplified Fragment Length Polymorphism (AFLP) analysis (VIDISCA) was performed as described by van der Hoek et al. [8], with minor modifications. Ten millilitres of culture supernatant were cleared by centrifugation at 8000 g. Supernatant thereof was centrifuged at 38.000 g for 4 h. Pellets were treated with 2 U of DNase 1 (Ambion) in 1X buffer, 100 μl reaction volume, at 37°C for 1 h. After phenol-/chloroform-based nucleic acids extraction, cDNA synthesis primed by random hexamer oligonucleotides was performed with the Superscript double stranded cDNA synthesis kit as recommended by the manufacturer (Invitrogen, Karlsruhe, Germany). Enzymatic digestion of cDNA involved HinP1I, as in the original protocol, and CviAII instead of MseI in order to optimise the 3'-end of the primer used for amplification later on. After digestion, 600 nM oligonucleotide linkers for the HinP1I-site (GACGATGAGTCCTGAT and Phosphate-CGATCAGGACTCAT, 1:1) and the CviII-site (CTCGTAGACTGCGTACG and Phosphate-ATCGTACGCAGTC, 1:1) were ligated to the complete phenol-purified digestion product with T4 ligase (Roche, Mannheim, Germany). The first amplification stage (20 cycles, 50 μl reaction volumes) used 300 nM of primers CTCGTAGACTGCGTACGATG and GACGATGAGTACTGATCGC at 56°C annealing temperature with Platinum Taq polymerase (Invitrogen, Karlsruhe, Germany). Second round amplification used four variants of each of the aforementioned primers, containing single nucleotide extensions of A, T, G, or C, respectively, at their 3'-ends. The resulting 16 different combinations of forward and reverse primers were each used on 2 μl of the first stage PCR product, under a touchdown cycling protocol: 95°C, 4 min, followed by 10 cycles of 94°C/30s, 65°C, 30s (temperature decrease of 1°C per cycle), 72°C 1 min, followed by 25 cycles of 94°C/30s, 56°C, 30s, 72°C, 1 min. Enzymes were the same as before. Products were analysed by agarose gel electrophoresis. Sequencing was done directly from second stage VIDISCA products on a Beckman 2000 XL system using the respective amplification primers.

### Sequencing of full genome

Partial P1 sequences were generated directly from the VIDISCA product. The full P1 sequence was determined using upstream primers in the 5'-noncoding region and a downstream primer in the VIDISCA product. The 2C- to 3D protein gene sequence was obtained by amplifying the highly conserved distal segment of the 3D gene. Matching candidate forward primers in the 2C protein were derived from an alignment of all prototype strains available in GenBank in November 2005. Long-range PCR was done with the Expand High Fidelity kit (Roche, Penzberg, Germany). Obtained products from successful long-range amplifications were cloned in pCR4 vectors (Invitrogen) and sequenced by primer walking technique. Primers in the P1 and the 2C/3D fragment were designed specifically for determined sequences and used to amplify a P1-2C fragment, which was also cloned and sequenced by primer walking. Sequences were confirmed from virus RNA by direct sequencing after sequencing of clones. All primer sequences are available upon request. GenBank accession number of strain BNI-788st is EF051629.

### Phylogenetic and recombination analysis

Phylogenetic analyses were conducted in MEGA4 [42]. RNA secondary structure was predicted by Mfold, version 3.2 (Zucker, 2003. Similarity plots and Bootscan analyses were done with Simplot software [43]).

## Abbreviations

AFLP: Amplified fragment length polymorphism analysis; VIDISCA: Virus Discovery cDNA AFLP; HPeV: Human parechovirus.

## Competing interests

The author(s) declare that they have no competing interests.

## Authors' contributions

LKSL conducted VIDISCA experiments and full genome sequencing.

SB did cell culture screening of stool samples and identified initially the described virus strain.

KG, MP, and JFD conducted full genome sequencing and assisted with in-silico analyses.

CD organised the work, conducted in-silico analyses, and wrote the manuscript. All authors have read and approved the final manuscript.
